# Epigenetic pace of ageing estimation in children and adolescents with and without childhood maltreatment and psychopathological symptoms: evidences from DunedinPACE clock

**DOI:** 10.1192/j.eurpsy.2026.10551

**Published:** 2026-07-31

**Authors:** N. San Martín-González, M. Acosta-Díez, D. Czamara, L. Marqués-Feixa, S. Romero, H. Palma-Gudiel, M. Rapado-Castro, I. Zorrilla, H. Blasco-Fontecilla, B. Arias, S. Papiol, L. Fañanás

**Affiliations:** 1 Department of Evolutionary Biology, Ecology and Environmental Sciences, University of Barcelona, Barcelona; 2 Centre for Biomedical Research Network on Mental Health (CIBERSAM), Madrid; 3 Biomedicine Institute of the University of Barcelona (IBUB), Barcelona, Spain; 4 Department Genes and Environment, Max Planck Institute of Psychiatry, Munich, Germany; 5 Department of Child and Adolescent Psychiatry and Psychology, Institute of Neuroscience, Hospital Clínic de Barcelona; 6 Institut d’Investigacions Biomediques August Pi i Sunyer (IDIBAPS); 7 Barcelonaßeta Brain Research Center (BBRC), Barcelona; 8 Department of Child and Adolescent Psychiatry, Institute of Psychiatry and Mental Health, Hospital General Universitario Gregorio Marañón; 9 School of Medicine, Universidad Complutense, IiSGM, Madrid, Spain; 10 Department of Psychiatry, Melbourne Neuropsychiatry Centre, The University of Melbourne & Melbourne Health, Victoria, Australia; 11 Department of Psychiatry, Hospital Santiago Apostol, Vitoria; 12 Department of Psychiatry, Puerta de Hierro University Hospital-Majadahonda , ITA Mental Health, Madrid; 13 Instituto de Investigación, Transferencia e Innovación, Ciencias de la Salud y Escuela de Doctorado, Universidad Internacional de La Rioja, Logroño, Spain; 14 Institute of Psychiatric Phenomics and Genomics (IPPG), University Hospital, LMU Munich; 15 Department Clinical Translation, Max Planck Institute of Psychiatry, Munich, Germany

## Abstract

**Introduction:**

Childhood maltreatment (CM) and mental disorders are often chronic conditions associated with poor health outcomes and increased mortality. Recent research indicates that those stressful conditions may accelerate biological ageing by means of epigenetic age acceleration. Little is known about how the exposure to CM and psychopathological symptoms contribute to biological ageing in children and adolescents recently exposed to such conditions.

**Objectives:**

i) to examine whether CM and psychopathological internalizing/externalizing symptoms impact the pace of ageing, and ii) to study the putative sex differences.

**Methods:**

203 children and adolescents (7 to 17 y.o.) from the Epi_Young_Stress project were included. DNA methylation in PBMCs was quantified using the Illumina EPIC_array_v1. Epigenetic pace of ageing was estimated using DunedinPACE clock. CM was evaluated by TASSCV and internalizing/externalizing symptoms with CBCL. Linear regression models were used to test associations between epigenetic ageing, CM and internalizing/externalizing symptoms. Age, socioeconomic status (SES), ancestry and smoking were included as potential confounders.

**Results:**

Lower SES was strongly associated with an accelerated pace of ageing. Stratified analyses revealed sex-specific effects: among females, SES was the primary predictor of increased ageing, while neither CM nor internalizing/externalizing symptoms exerted significant effects. Among males, externalizing symptoms and smoking were significantly associated with an increased pace of ageing.

**Conclusions:**

SES emerged as the strongest determinant of epigenetic ageing in children and adolescents. In males, externalizing behaviors and smoking further contributed to accelerated ageing, underscoring the relevance of lifestyle and psychosocial wellbeing in shaping ageing trajectories early in life.

**Disclosure of Interest:**

None Declared

